# Supplementary material for: Volcaniclastic density currents explain widespread and diverse seafloor impacts of the 2022 Hunga Volcano eruption
Source: Nat Commun. 2023 Nov 30;14:7881. doi: 10.1038/s41467-023-43607-2 (PMC10689732; doi:10.1038/s41467-023-43607-2)
Supplement: Supplementary file 1 — Supplementary Information [file 41467_2023_43607_MOESM1_ESM.pdf]

Table S1: Thickness of deposits related to the Hunga Volcano eruption sampled at the different multicore sites, including identification of deposits attributable to the volcaniclastic density currents and to ashfall. Thickness annotated as >X cm indicate that the core did not recover the base of the deposits and that the true thickness is greater than that presented. Station numbers are given in Figure S8.

Core Site (Station # / Site #)	Latitude	Longitude	Total thickness of HTHH deposits sampled [cm]	Density current deposit thickness [cm]	Ashfall deposit thickness [cm]
090/23	-20.9525	-176.039	30.5	23	7.5
096/31	-21.1357	-176.131	48.5	47	1.5
022/16	-20.7047	-175.292	42.5	42	0.5
087/21A	-20.7338	-175.64	>18.5	>12.7	5.8
087/21B	-20.7338	-175.64	>19.0	>15.9	3.1
083/4	-20.9177	-175.647	>22.0	>19.0	3.0
065/30	-20.3915	-175.572	>32.2	>27.7	4.5
095/24	-21.0385	-176.323	6.5	4.5	2.0
070/17	-20.8123	-175.267	>11.2	>11.2	Absent
031/20	-20.6763	-175.532	>33.8	>32.8	1.0
036/22	-20.8772	-175.899	38.0	34.0	4.0
076/18	-20.916	-175.199	2.45	2.45	Absent

Table S2: An overview of runs undertaken for volcaniclastic density current model.

	Model Runs	Initial conditions	Differences observed
1	Different volumes $2m^3$, $3km^3$ and $4km^3$ of initial density current.	Initiated with a column of dense fluid located in caldera, 4 km radius, height as required for volume (40, 60, 80 m)	Density current reaches further for larger volumes
2	Changes in density of the density current ($1{,}400kg/m^3$ and $1{,}800kg/m^3$ were tested in addition to $1{,}600kg/m^3$)	As for model 1 with different density for density current	Small, second order changes in distribution of the density current.
3	Changes to the friction of the density current (Higher friction coefficients of 10^{-3} and 10^{-2} in addition to 10^{-4})	As for model 1 with different friction for density current	Density current does not reach as far for higher friction coefficients.
4	Simple representation of crater rim without the islands Hunga Tonga and Hunga Ha'apai Vs reconstruction of crater rim from post-survey bathymetry including the remnants of Hunga Tonga and Hunga Ha'apai and the dip in the crater rim	As for model 1 with different bathymetry on top of the Hunga Volcano Edifice	The full reconstruction of the crater rim channeled a significant part of the density current northwest down the channel where significant erosion was observed. Without this bathymetry, it flowed directly northwards.
5	Initiation of density current located outside of the crater (i.e., to represent a fountaining PDC flowing into the ocean and forming a density current)	Initiated with an annulus of dense fluid between 5 and 7 km radius from the caldera centre (i.e., outside of the caldera), 53 m high ($4km^3$ volume in total), and an inital radial velocity of 10 m/s.	The fountaining initiation did not channel the density current down the channel where erosion was observed.

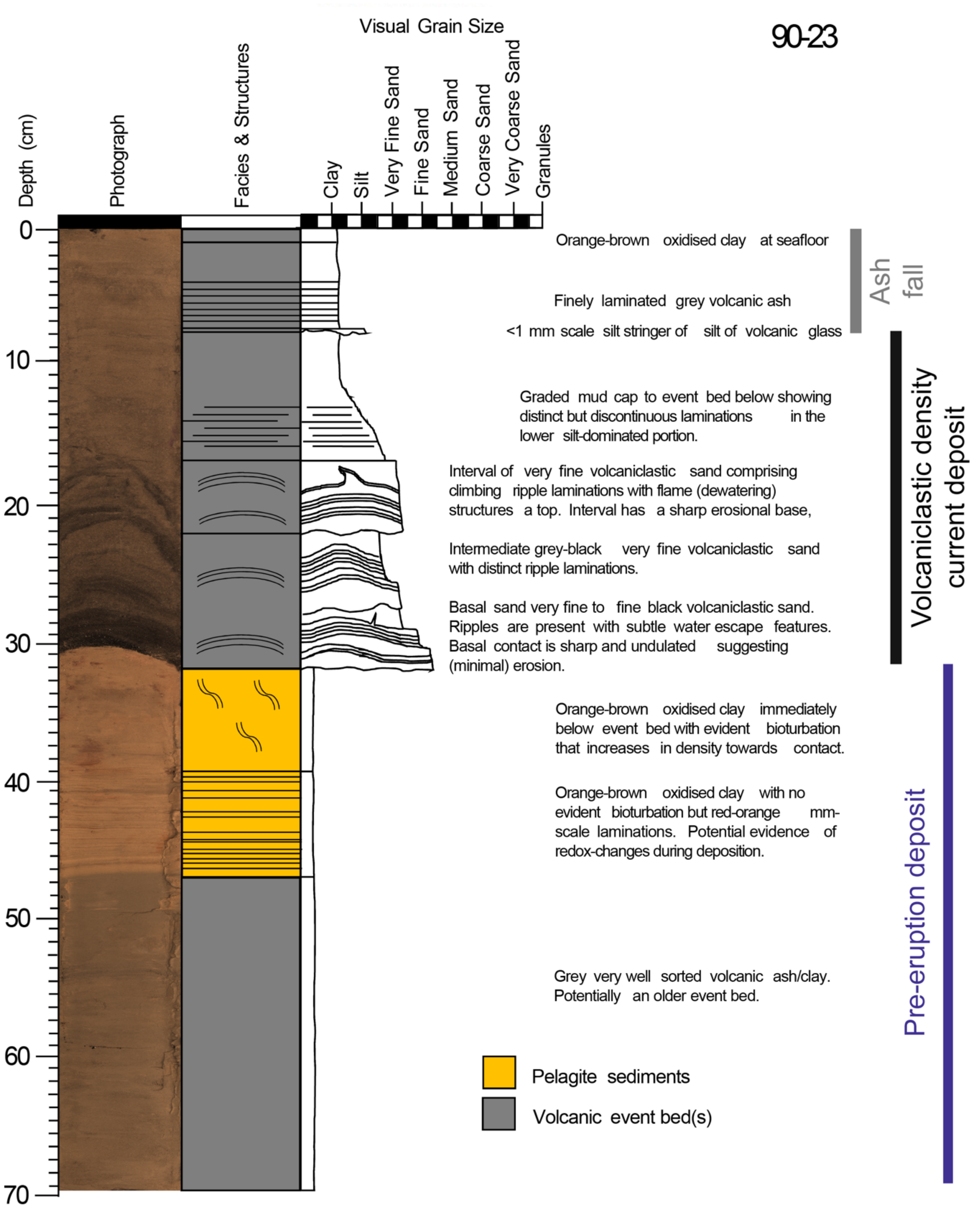

Figure S1: Representative visual core log from multicore location 90/23 (80 km from caldera), illustrating pre-eruption, volcaniclastic density current and ash fall deposits.

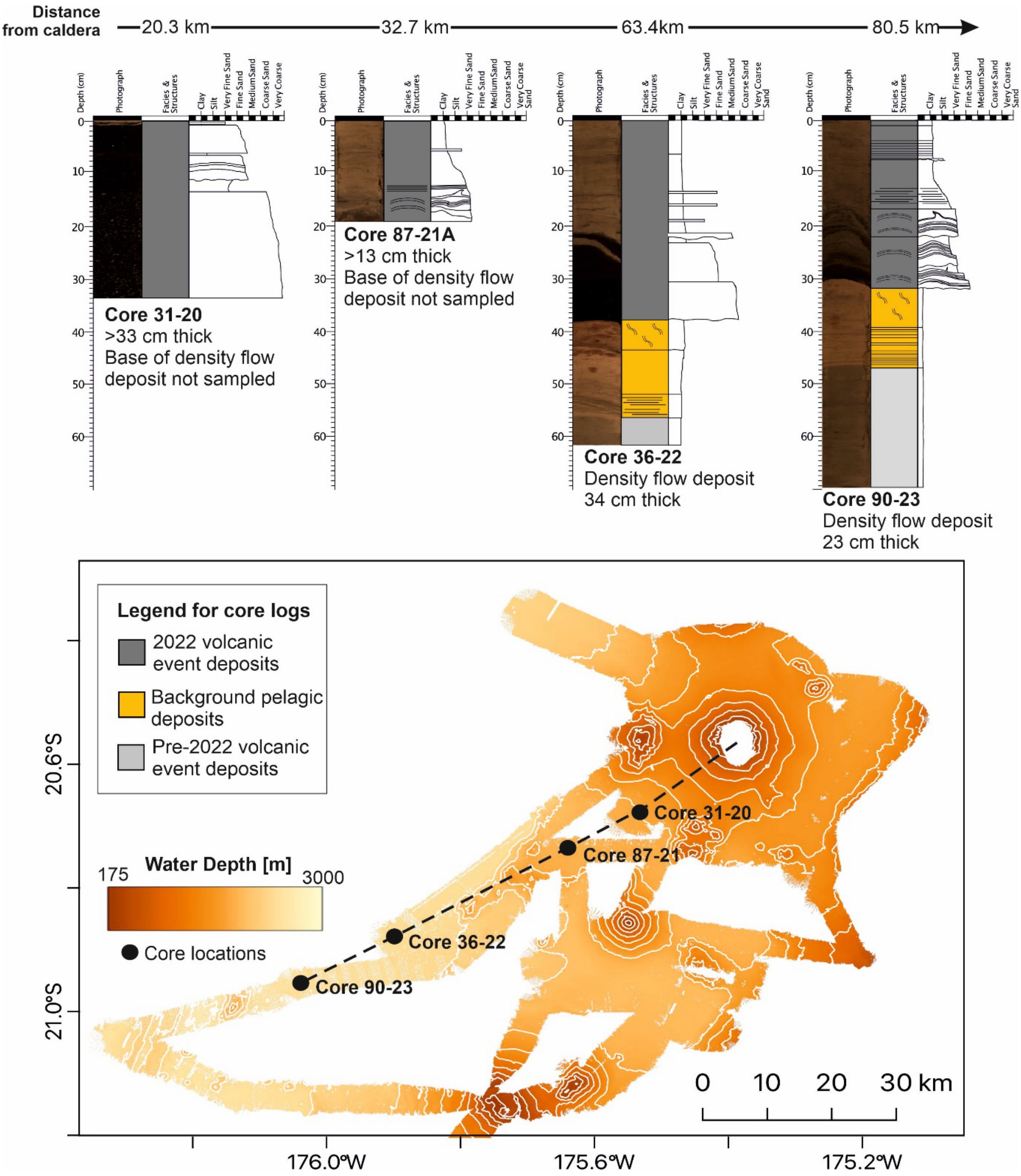

Figure S2: Representative visual core logs from multicore location along southwest transect from caldera, illustrating pre-eruption, volcaniclastic density current and ash fall deposits.

Expanded Geochemical Results (See Supplemental Data 1 for complete data)

Effect of washing submarine ash on major element composition.

The major element composition of sample 90-1 varied depending on the extent of washing the sample had undergone (Figure S3, Supplementary Data 1). After five days of washing Na_2O contents had almost halved, going from 3.47 wt % in the unwashed sample to 1.86 wt. %. Similarly, Cl and S were excessively high in the unwashed sample (1.4 wt. % and >900 ppm, respectively) although equilibrium was reached after 2 days of washing (~ 0.1 wt. % and ~500 ppm). Due to the high contents of these elements in unwashed samples (and samples only washed for a day) other elements had correspondingly lower totals. The most pronounced effect was on SiO_2, which was ~1.6 wt. % in the fully washed sample compared to the unwashed sample.

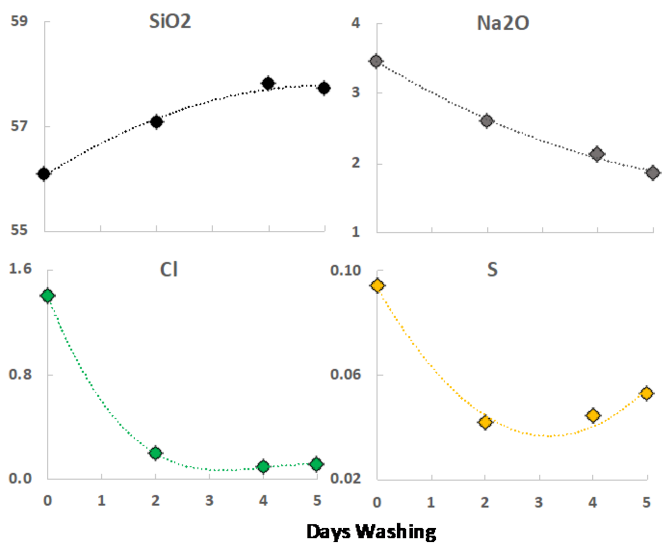

Figure S3: The effect of washing ash samples in distilled water on the chemical composition. Initial Na_2O, Cl and S values were high in unwashed samples but plateaued after 5 days washing. Conversely SiO_2 significantly increased.

These results, for fine ash samples, are consistent with a previous study on whole rock samples undertaken by Tani et al. (2003) and highlight the importance of adequately washing submarine samples to remove seawater contaminants.

The source of ash in TAN2206 cores.

All core samples analysed are andesitic in composition. Glass shards from ash collected from Tongatapu and those found in CTD casts, overlap in composition with core samples, but range to dacitic in composition. This may be due to higher degrees of fractionation of the glasses, or an artefact of the semi-quantitative nature of the EDS analyses. Nevertheless, all samples have compositions similar to samples from HT-HH reported by Brenna et al. (2022) and are distinct from samples analysed from other nearby volcanoes. This is consistent with the TAN2206 samples originating from the 2022 HT-HH eruption, as indicated by seafloor bathymetry and images.

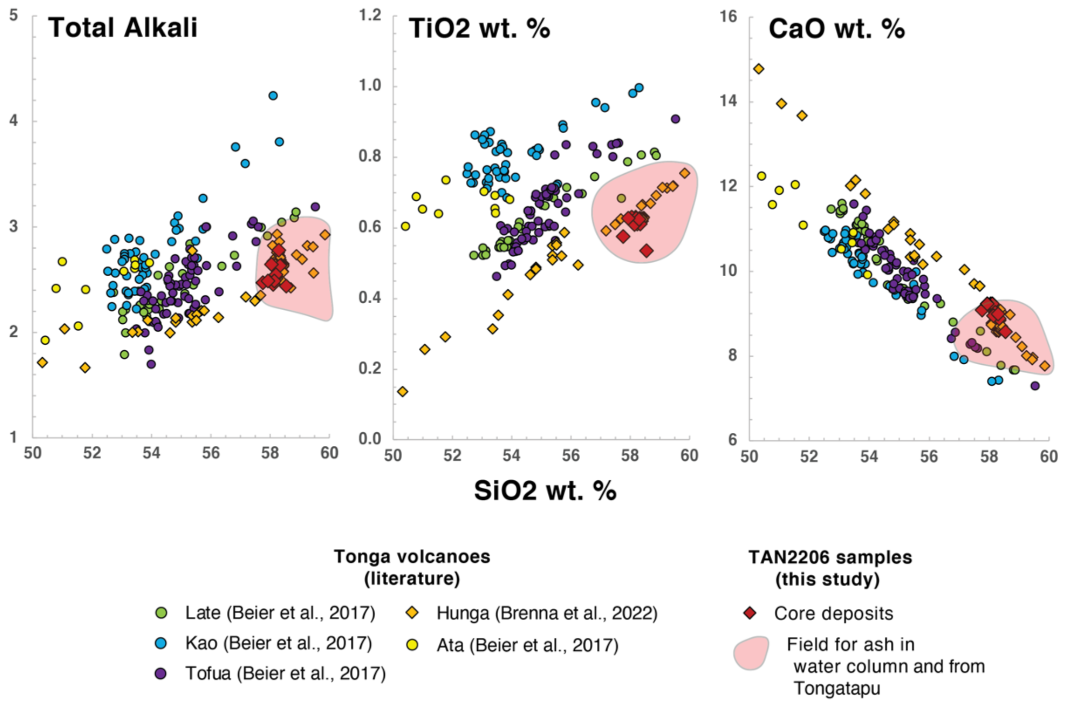

Figure S4: **Major element compositions of core material (T2206 ash) and glass shards from TAN2206 and ash from Tongatapu (T2206 shards) compared to previously published data from HT-HH (Brenna et al., 2022) and surrounding volcanoes: (north to south) Late, Kao, Tofua and Ata (Beier et al., 2017).**

Core samples have a more restricted compositional range than samples from HT-HH analysed by Brenna et al. (2022), which can be explained by their petrological composition. The Brenna et al. (2022) samples are whole rock compositions that show extensive fractionation, whereas TAN2206 samples are mostly fine ash, so are more representative of glass compositions than a mixture of glass and minerals. The exception are the two sandy samples (31-III and 70-Ash), which have a high proportion of felsic material (plagioclase), resulting in higher Al_2O_3 and lower $Fe2O_3$ and TiO_2 contents.

Samples from core 90, representing different depositional phases of the eruptive deposits, have similar compositions, suggesting little chemical variation between the depositional phases. Significantly, the old seafloor sample (90 base) is indistinct from other samples, indicating that previous eruptions from HT-HH also deposited material in this locality.

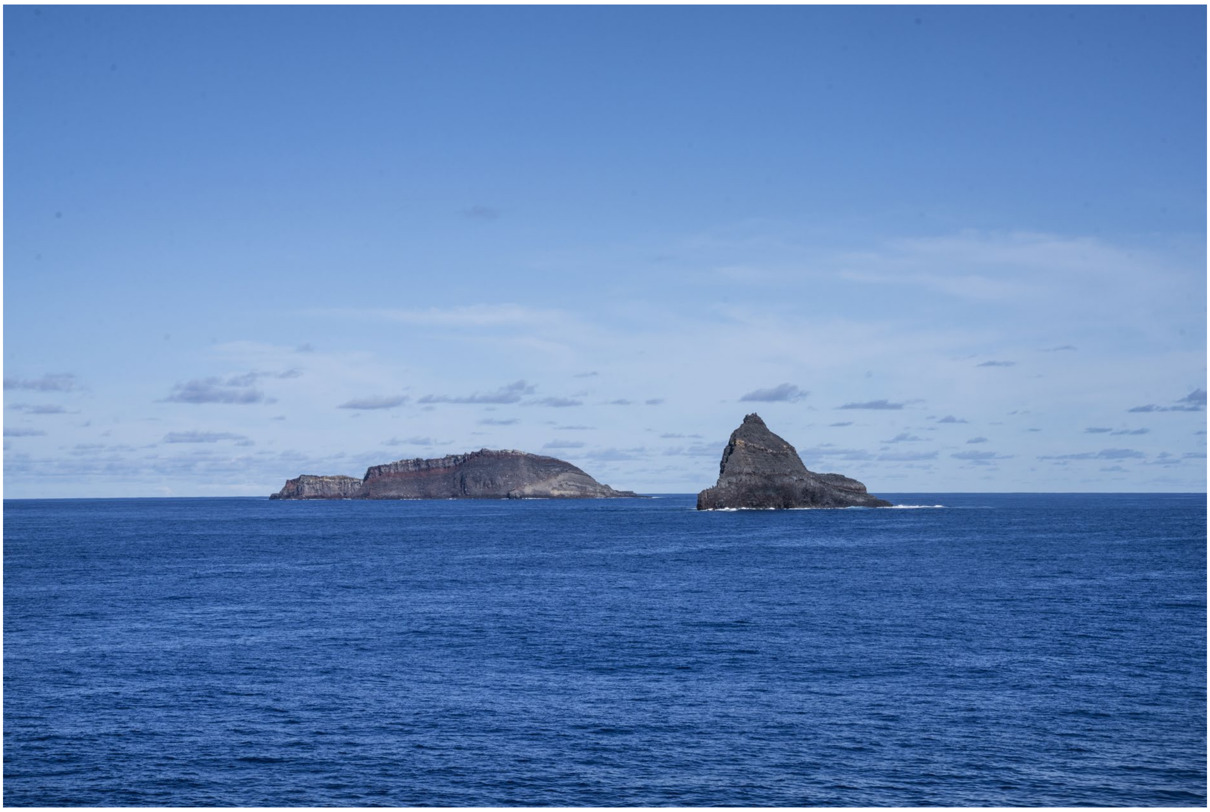

Figure S5: The remnants of the islands of Hunga Tonga and Hunga Ha'apai taken during the TAN2206 voyage three-months post eruption. Photo taken by NIWA-Nippon Foundation TESMaP / Rebekah Parsons-King

Sensitivity testing of density current model.

A variety of different configurations of the volcaniclastic density current model were simulated to test the sensitivity of the model. Because the model is a very simple one that only captures some aspects of the underwater volcaniclastic density current we need to be careful about how much we infer from these but they are instructive about points of the flow. Table S2 outlines the different models run and tthe results of some of these sensitivity tests are shown in Figure S7. These should be compared with the snapshots of the density current modelled in Figure S6. A fountaining initialisation of the volcaniclastic density current was simulated where the pyroclastic material mixes with the water outside of the caldera. Figure S7 (a) shows this model at time t=300s and should be compared with Figure S6 (a), these two figures are close-ups to highlight the differences. Figure S6 (a) clearly shows the density current flowing down the observed channels on the flanks of the Hunga edifice whereas this is not seen for the fountaining initialisation. This shows that whatever the mechanism that caused the density current, it had to occur inside the caldera because if it had not we would not see the channelisation of the flow and the resulting scour that was clearly observed. Figure S7 (b) shows the density current at t=2,400s for adensity current with initial volume of 2 km^3 of dense flow (which would be the equivalent of 0.8km^3 of 2,500 kg/m^3 density pyroclastic material mixed with water given our assumption of a density of 1,600 kg/m^3). In this model the flow barely makes it to the valley south of Hunga where the international cable lies and without enough momentum to cause significant damage. This shows that a

sufficiently large flow must have occurred at one time in order for it to reach this far. A series of smaller flows over the course of the eruption would not have reached as far. Figures S7 (c) and (d) test variations in the overall density of the flow and show model runs with lower (c) and higher (d) density at time t=2,400s. These show that the model is fairly insensitive to changes in density of the flow. In our main simulation the density is taken to be 1,600kg/m^3 which assumes that the flow is 40% (by weight) dense pyroclastic material and 60% water. The tests show that the flow does not reach quite as far for a less dense flow (1,400kg/m^3, 26.7% pyroclastic material, Figure S7 (c)) and reaches slightly further for a denser flow (1,800kg/m^3, 53.3% pyroclastic material, Figure S7 (d)) but in both cases the differences are fairly subtle.

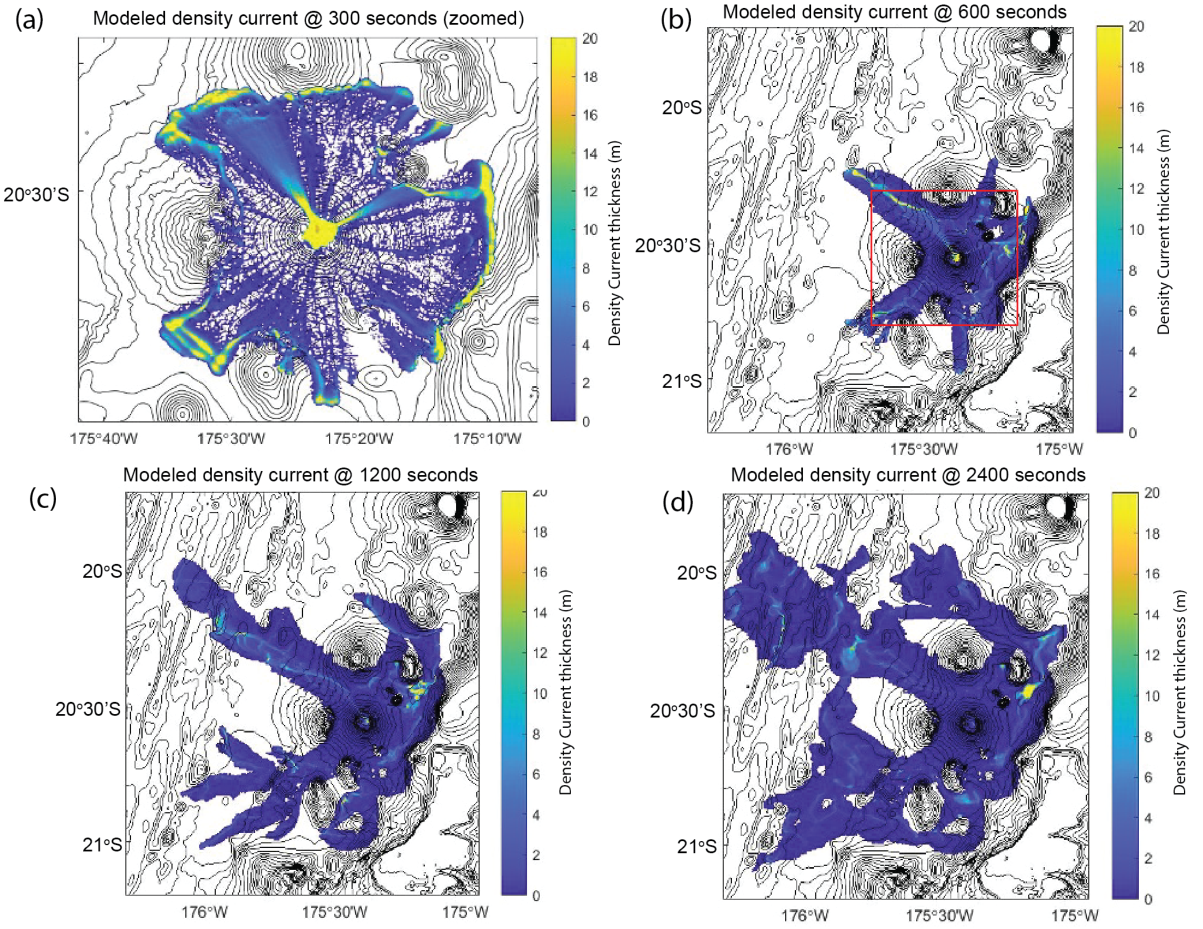

Figure S6: Select images of the Volcaniclastic Density Current model interpreted in Figures 2 and 3, and given in Supplemental Movie 1 (Model 1 in Table S2 with 4km^3 density current). (a) Close-up (red box in (b) shows extent of this image) of density current at 300 s (only density current shown for thickness greater than 5 cm is shown for comparison with the fountaining density current scenario shown in Figure S7 (a). (b) Density current at 600 s. Red box shows extent of Figures S6 (a) and S7 (a). (c) Density current at 1,200 s. (d) Density current at 2,400 s for comparison with S7 (b), (c) and (d).

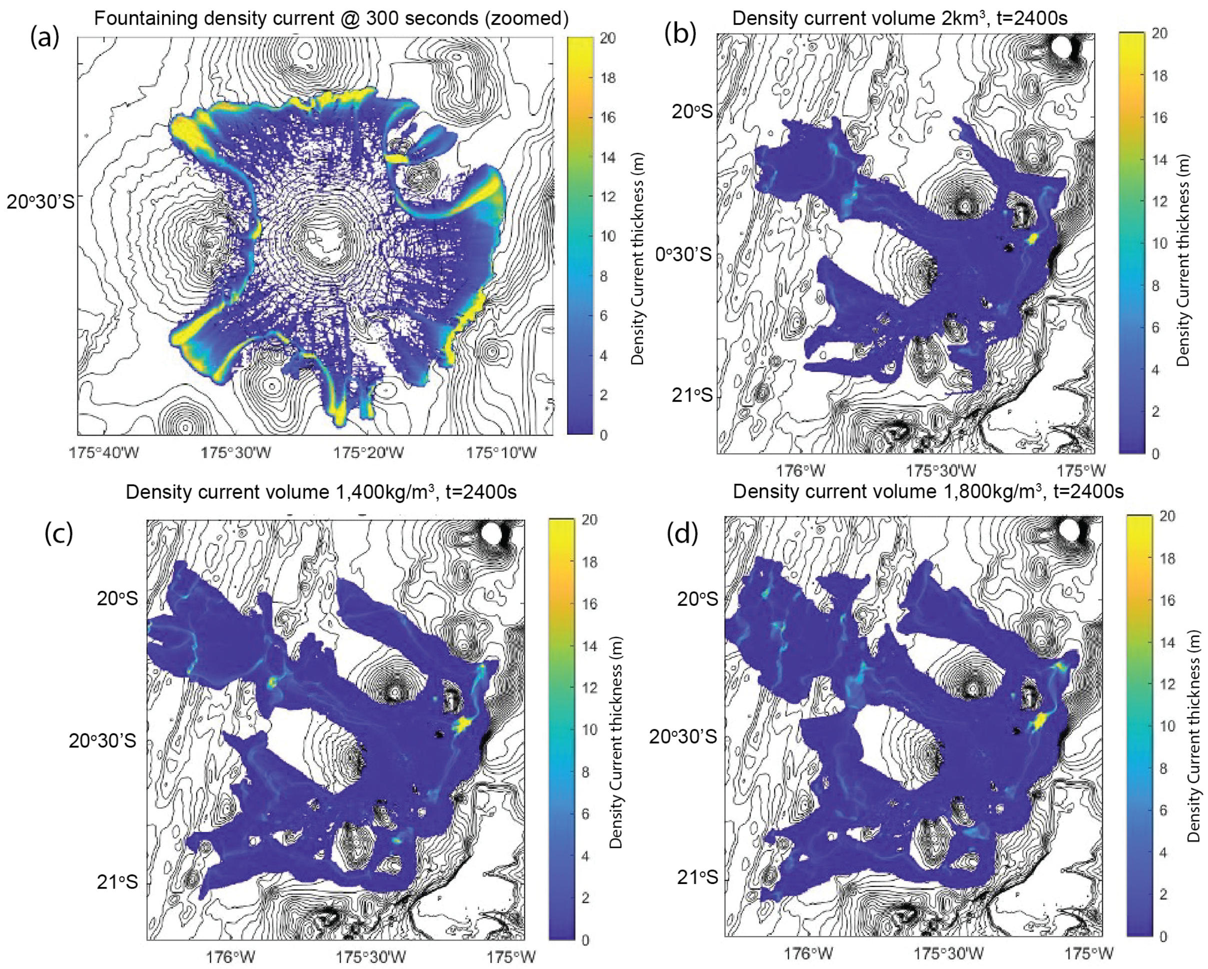

Figure S7: Sensitivity tested for volcaniclastic density current model for comparison with Fig. S6. (a) Close-up (red box in Figure S6 (b) of ffountaining initialisation (Model 4 in Table S2) at for thickness greater than 5cm (for comparison with Figure S6 (a)). (b) Density current with initial volume of 2km^3 (Model 1 in Table S2 with 2km^3 volume) at 2,400 s. (c) Density current with density of 1,400kg/m^3 (Model 2 in Table S2) at 2,400 s. (d) Density current with density of 1,800kg/m^3 (Model 2 in Table S2) at 2,400 s. (b),(c) and (d) should be compared with Figure S6 (d).

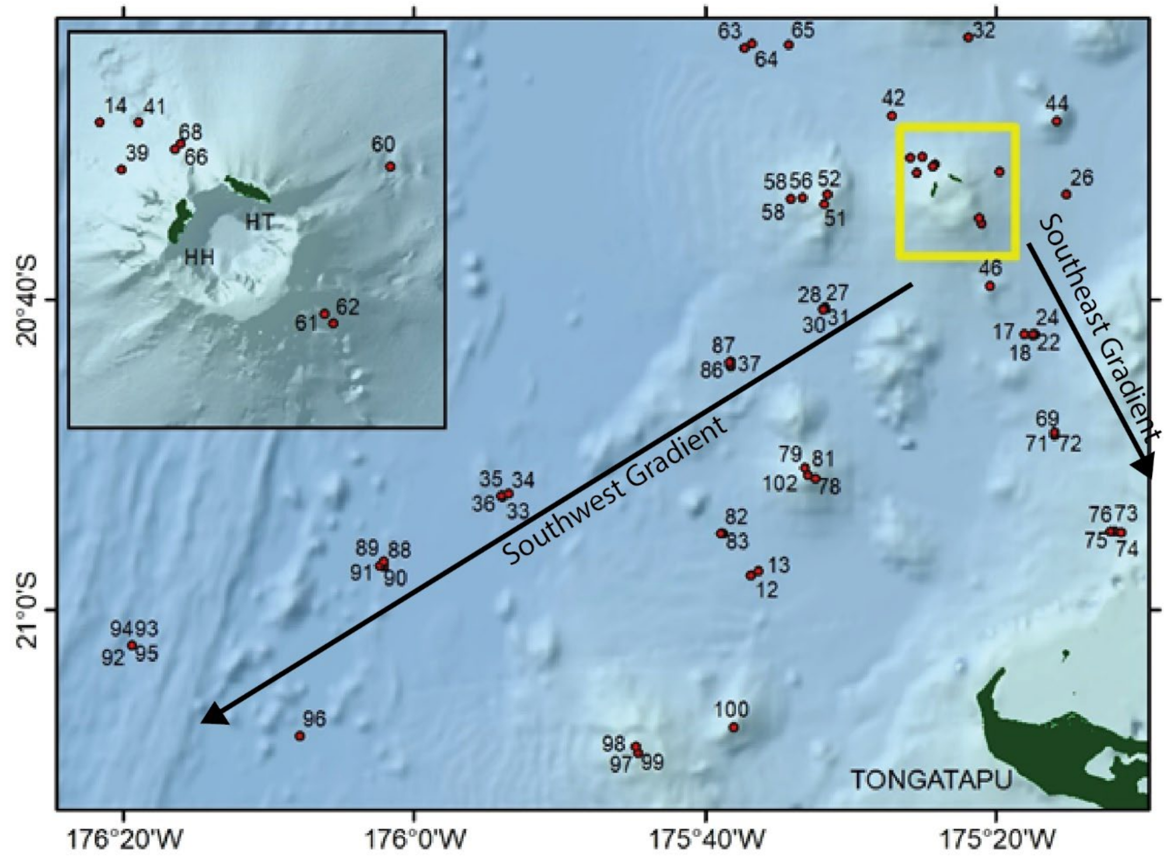

Figure S8: Map of study region with station numbers indicated, which correlate to the station numbers given in the included tables and figures below, as well as additional supplementary tables.

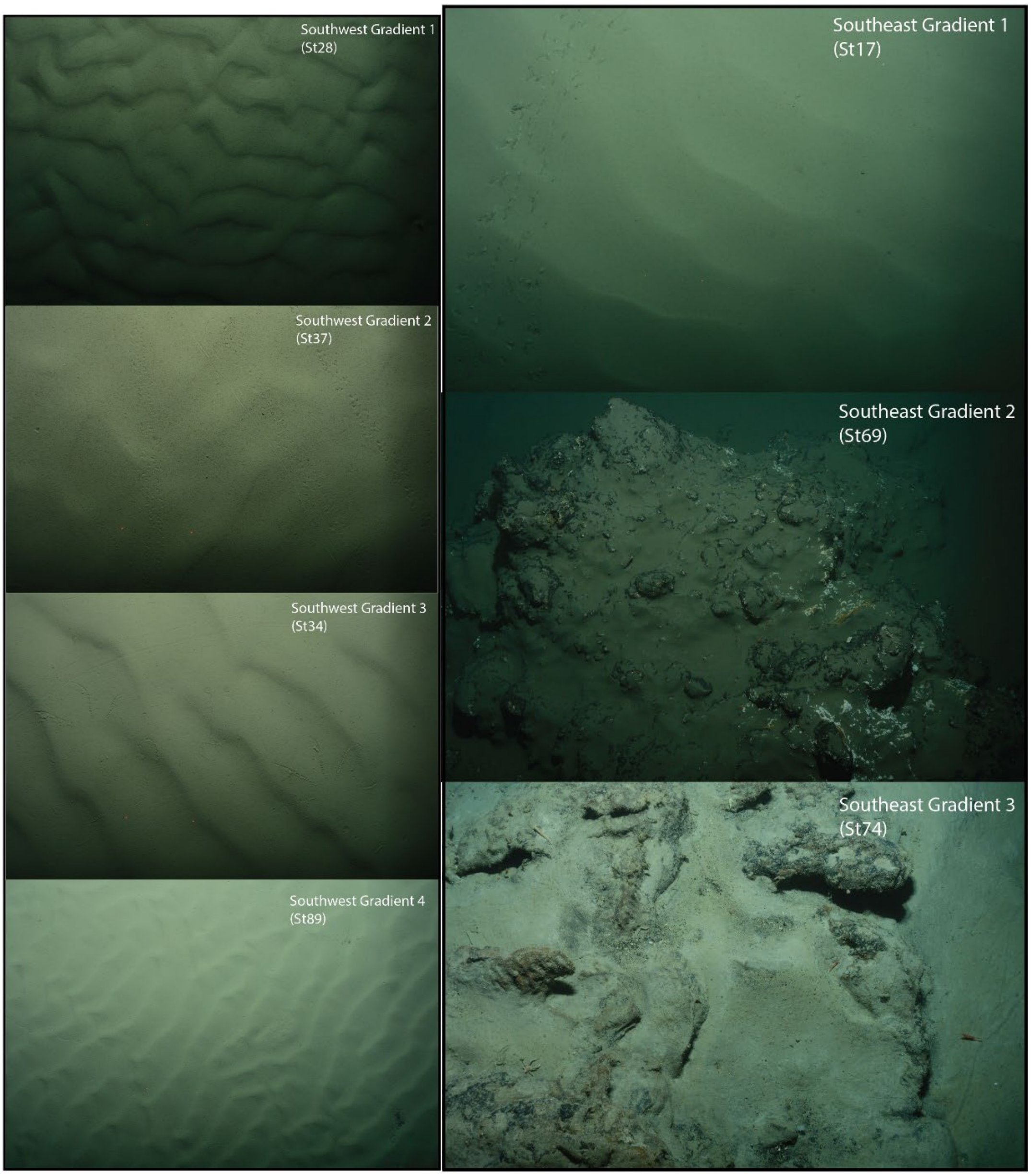

Figure S9: Images from the DTIS along the Southwest and Southeast gradient sites, with Station numbers correlated to Figure S8 indicated. These regions are interpreted as being regions of density current deposition. Seafloor ripples were obvious extending across both gradient sites, with fine ashfall over rocky terrain also seen.

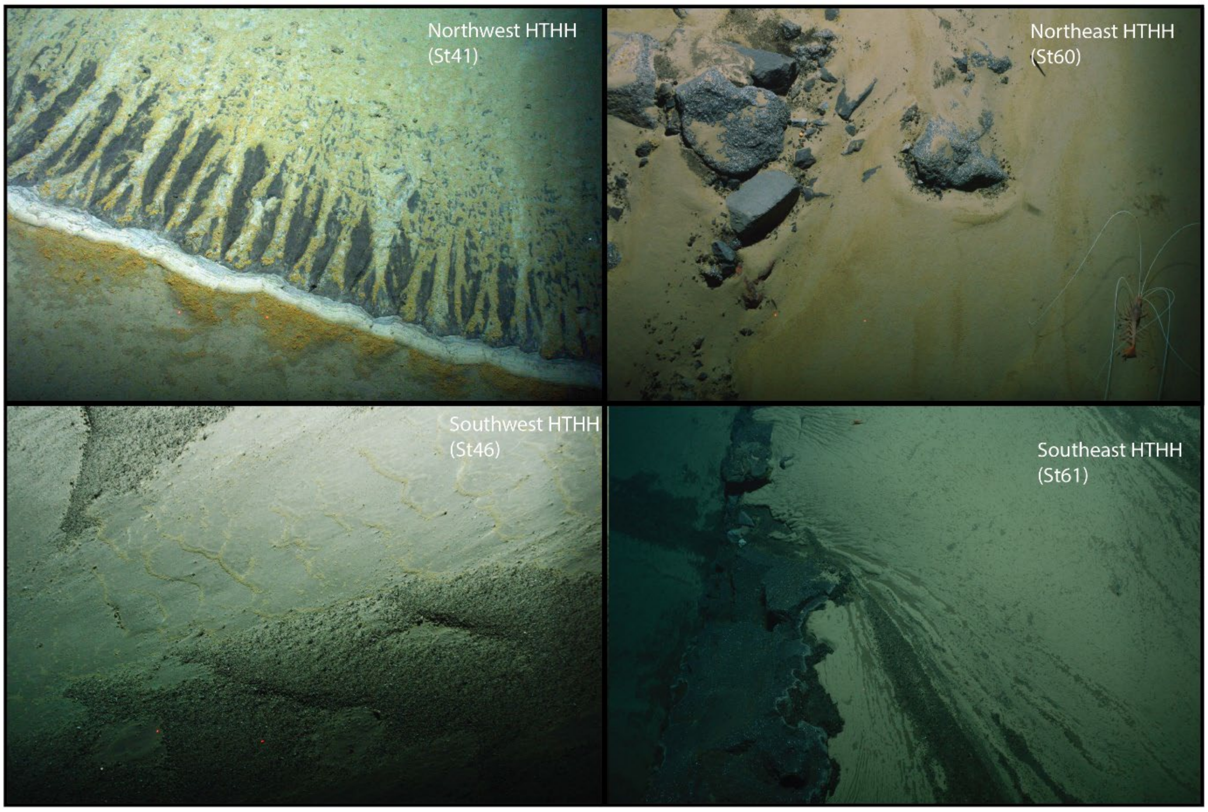

Figure S10: Images from the DTIS along the flanks of Hunga Volcano, with station numbers correlating to Figure S8 indicated.

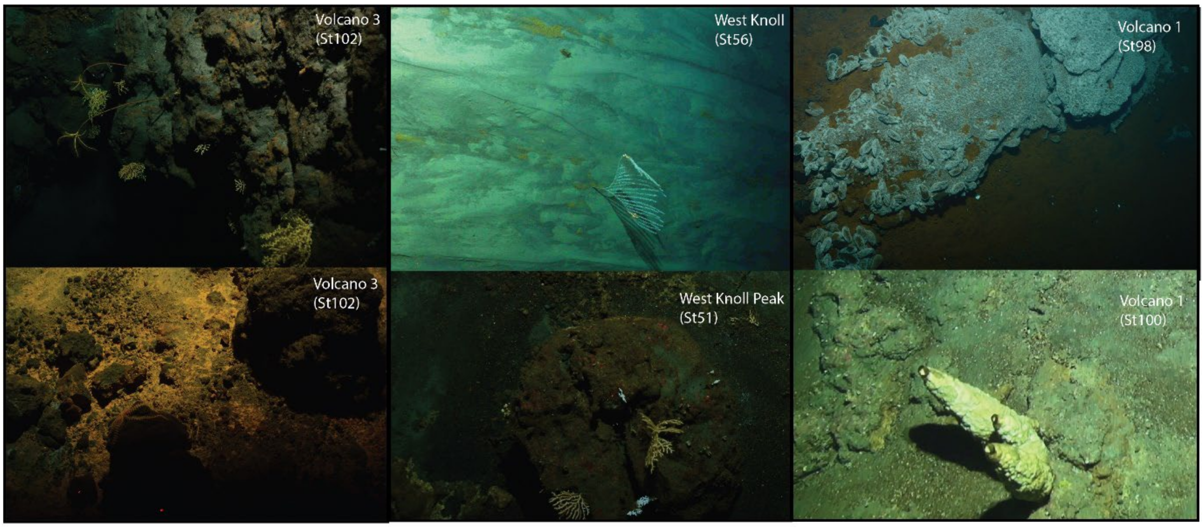

Figure S11: Seafloor images from DTIS from three seamounts in the region. Station numbers correlate to those indicated in Figure S8. Deep sea corals and other invertebrates can be seen, as well as microbial mats, the mussel bed found by R/V Sonne in 2007, and a chimney structure.

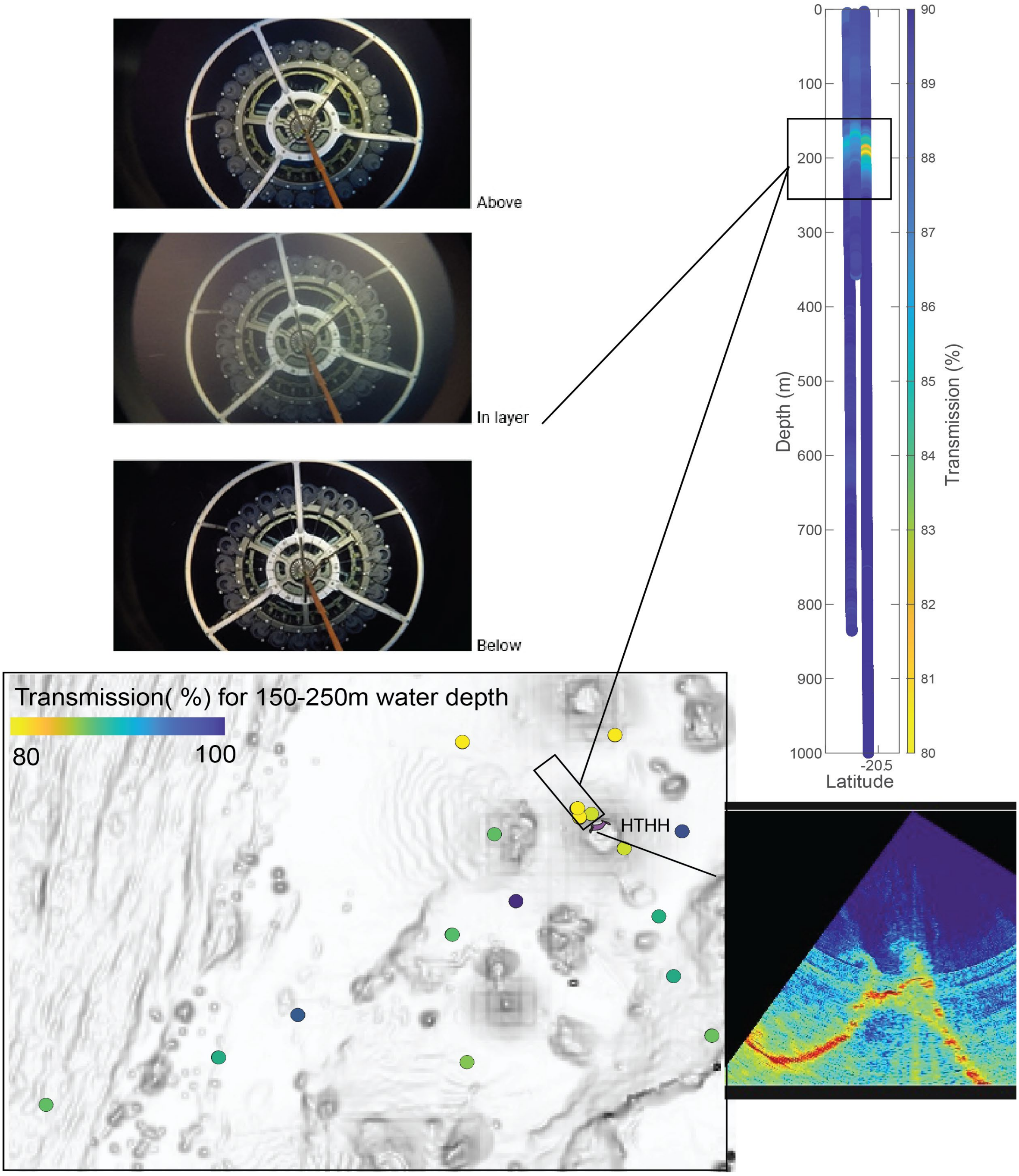

Figure S12: Top left: images of the CTD in the water column, above, within, and below the midwater ash layer. Top right: The corresponding high turbidity layer is shown with a vertical cross section of the water column, a lower % beam transmission corresponds to more particles in the water column. Percent beam transmission was measured with a transmissometer on the CTD rosette. Bottom left: a map of the region with dots coloured by the intensity of the beam transmission of the CTD. Bottom right: water column acoustics indicating a gas or particle plume on the caldera rim, with the location indicated, the nature of which is to be determined with future research (collected with the Kongsberg EM710, which has 65-100 kHz frequency).

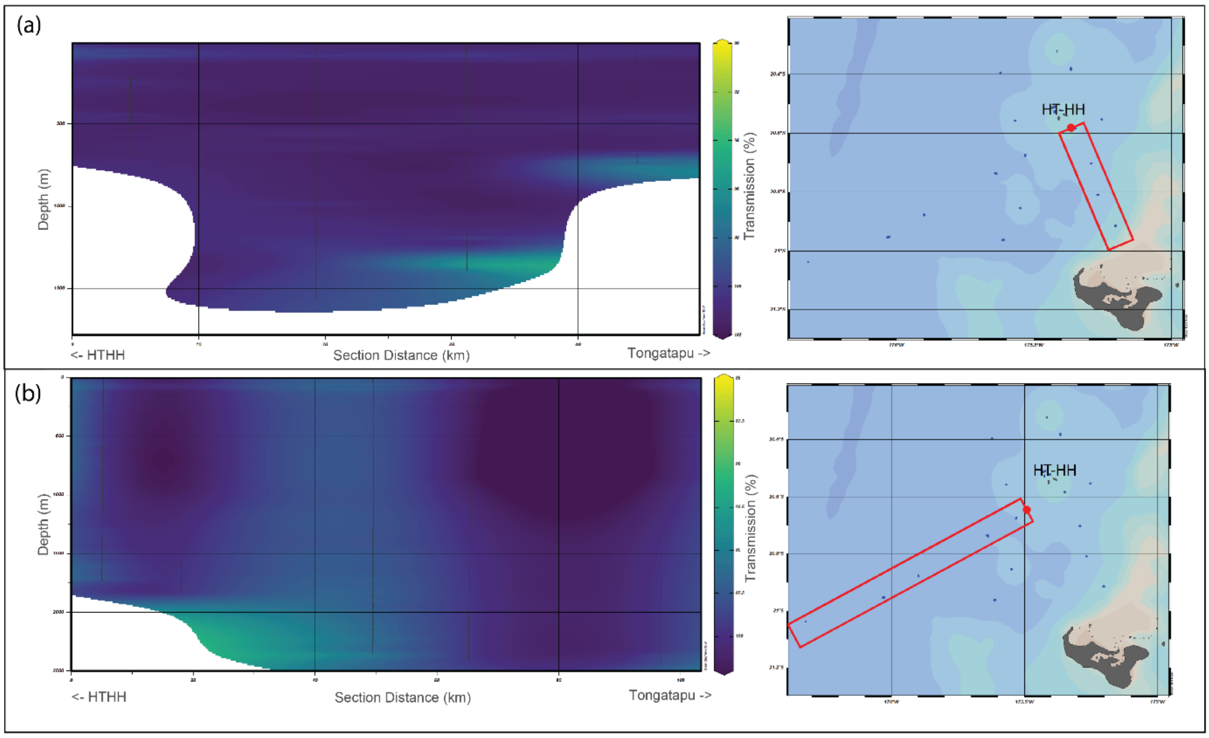

Figure S13: Percent beam transmission from the transmissometer on the CTD Rosette, gridded across the sections indicated in maps on the right. (a) is Southeast gradient, extending out from HTHH, and (b) is the Southwest gradient, extending out from HTHH.

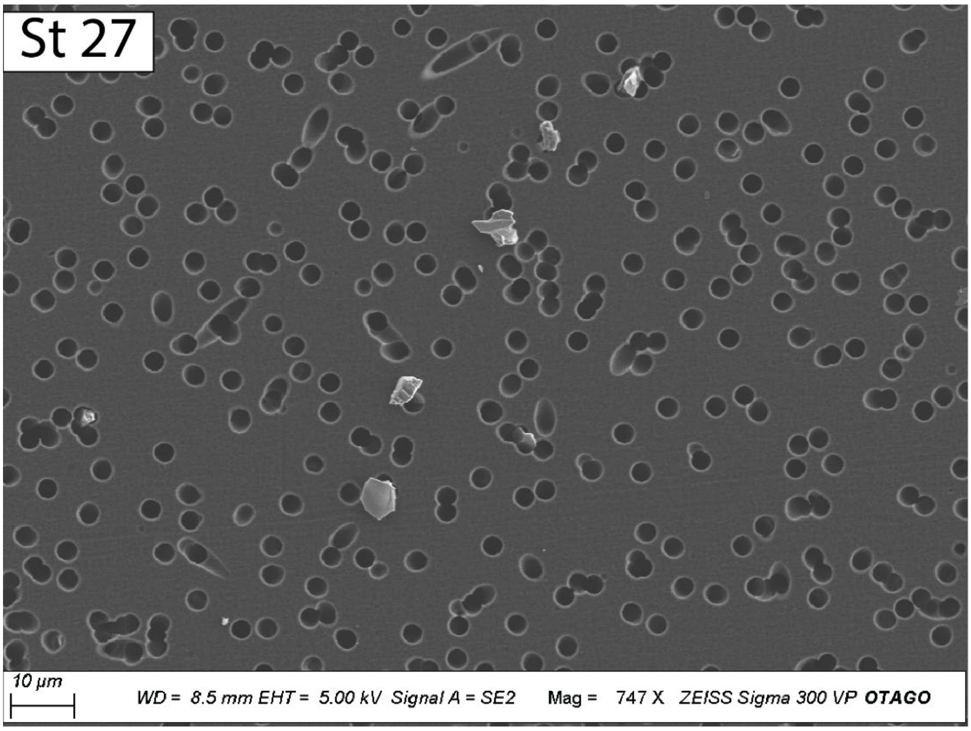

Figure S14: SEM image of ash particles from nepheloid layer at St 27, a site within the Southwest gradient shown in Fig. S11, and noted on the map with station number in Fig. S8

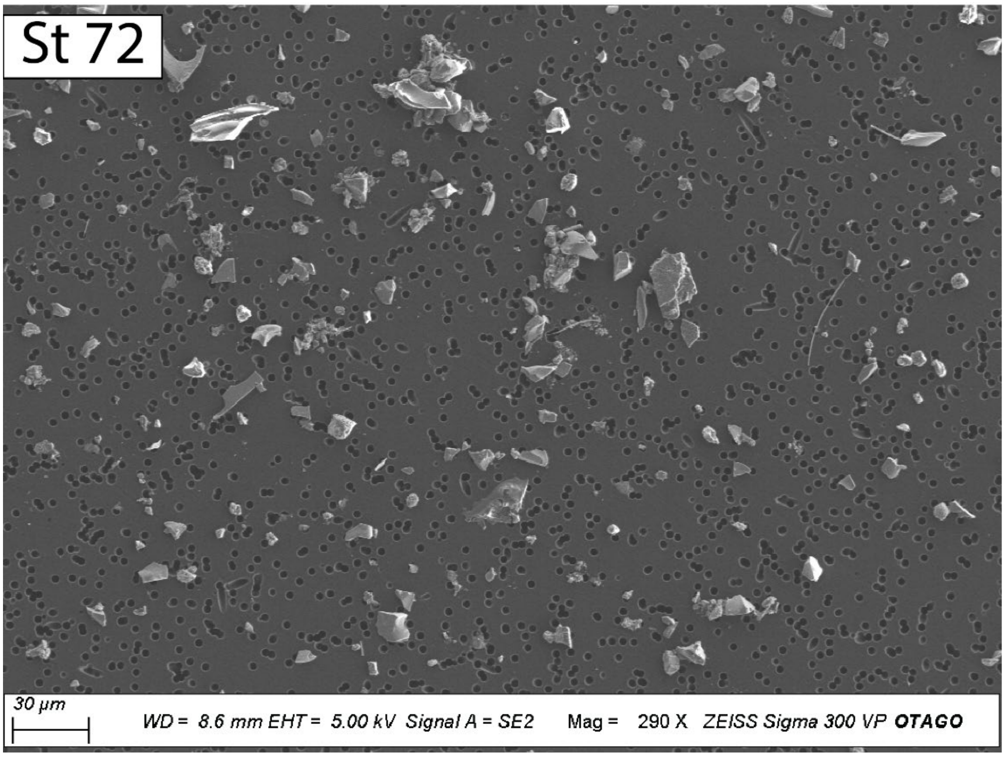

Figure S15: SEM image of ash particles from nepheloid layer at St 72, a site within the Southeast gradient shown in Fig. S11, and noted on the map with station number in Fig. S8

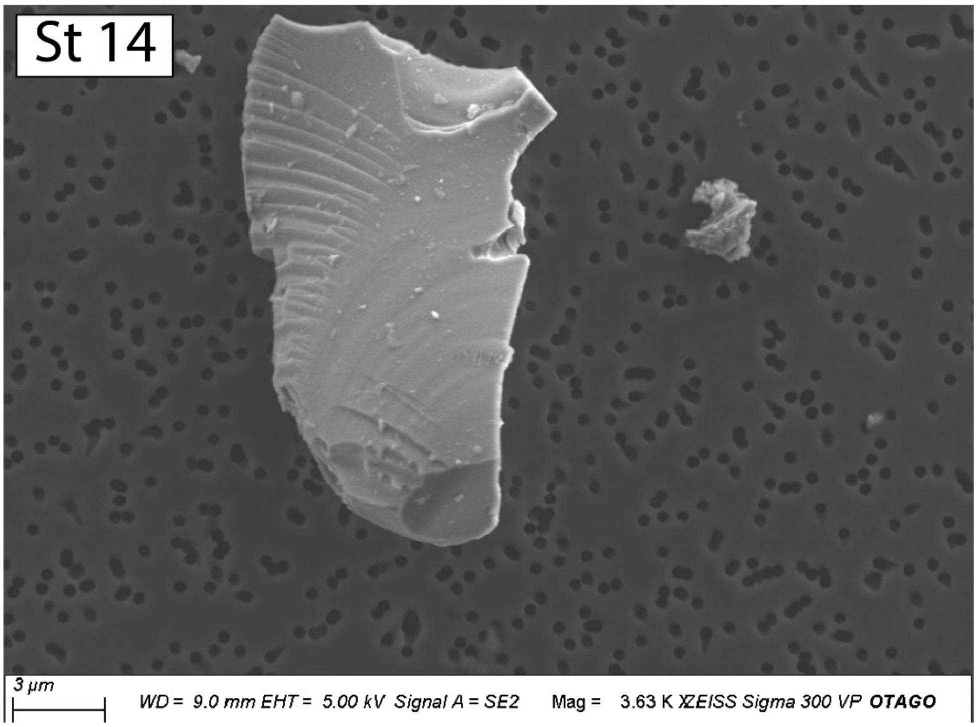

Figure S16: SEM image of ash particles from midwater ash layer at St 14, noted on the map with station number in Fig. S8

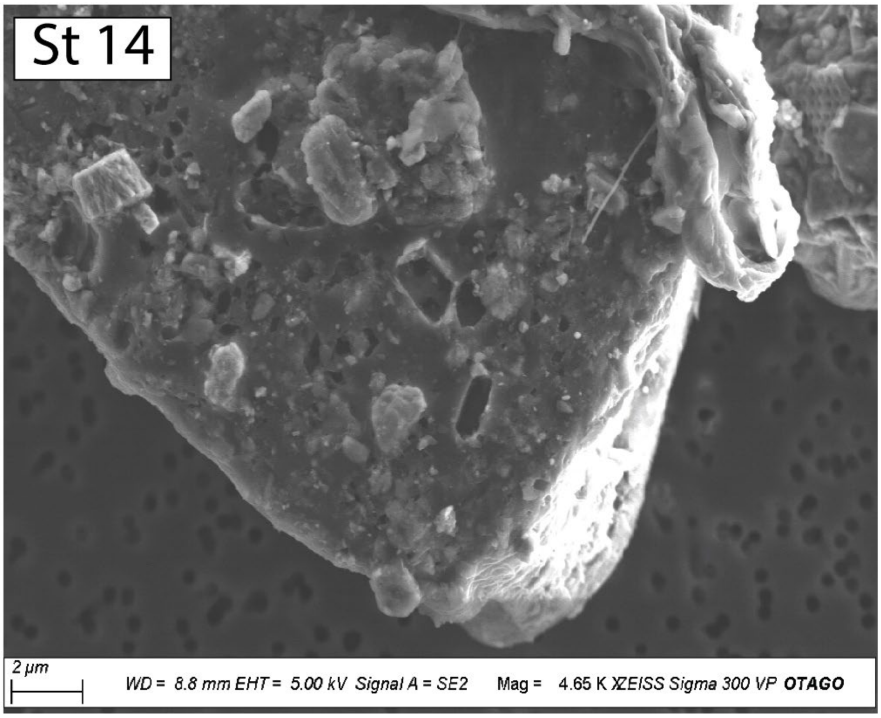

Figure S17: SEM image of ash particle from midwater ash layer at St 14, noted on the map with station number in Fig. S8

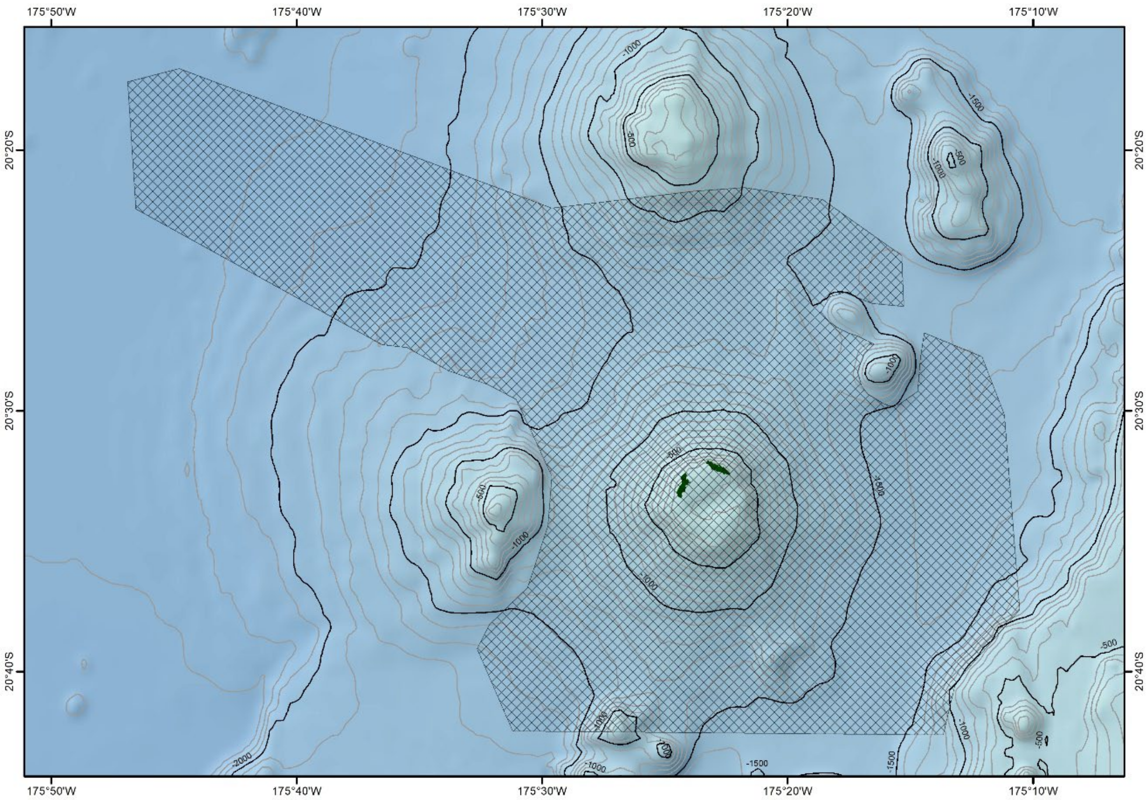

Fig S18: Polygon of region used for volumetric calculation of seafloor change following the eruption from the differences between pre- and post-eruption bathymetry.

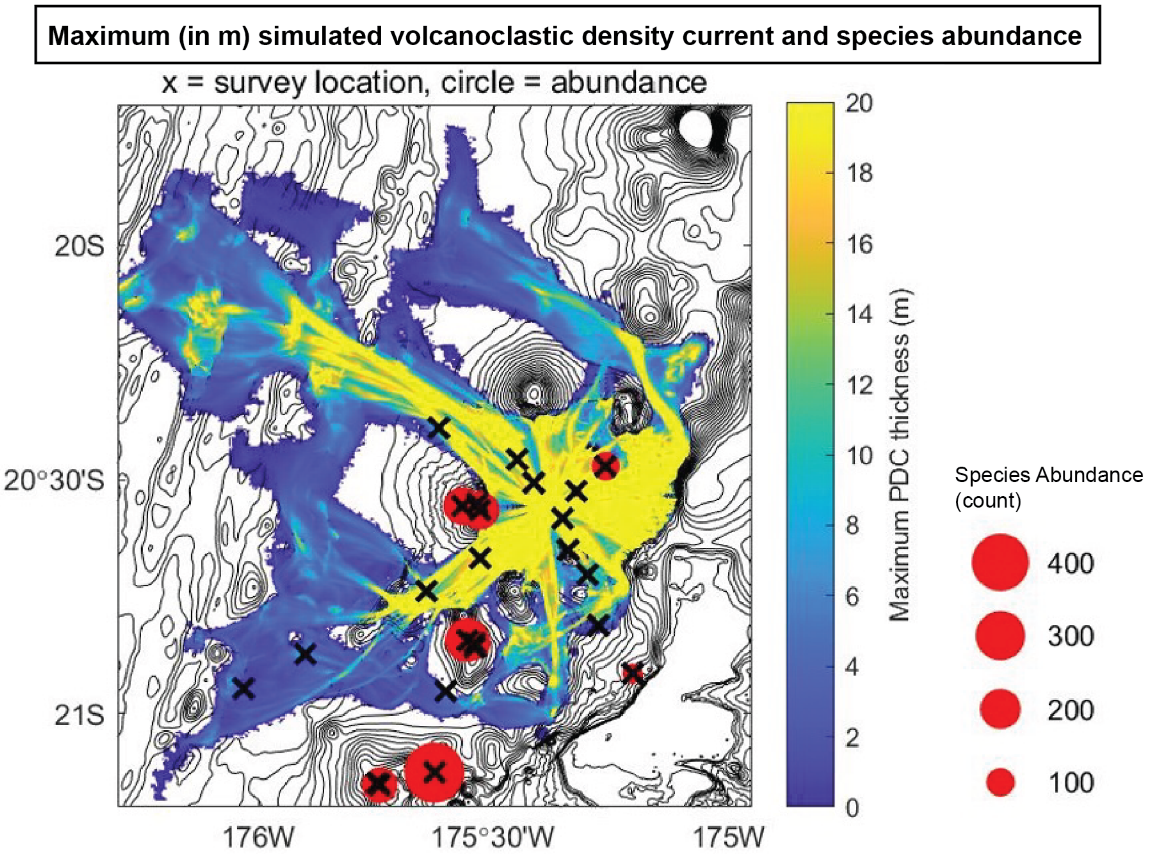

Figure S19: Comparison of maximum thickness of modelled volcaniclastic density current (model 1 in Table S2 for 4km^3 volume density current) over the course of the simulation and abundance of fauna found in survey. Black X's shows survey locations and size of red circles shows abundance of fauna found at that location (survey locations that show up as black X's with no red circles are areas where no fauna where observed; see Supplemental Data 2 and Fig. 3 for more reference).

References

1. Beier C., Turner, S.P., Haase, K.M., Pearce, J.A., Munker C., Regelous, M., 2017. Trace element and isotope geochemistry of the northern and central Tongan Islands with an emphasis on the genesis of high Nb/Ta signatures at the northern volcanoes of Tafahi and Niuatoputapu. Journal of Petrology 58, 1073-1106. doi: 10.1093/petrology/egx047
2. Brenna, M., Cronin, S.J., Smith, I.E.M., Pontesilli, A., Tost, M., Barker, S., Tonga'onevai, S., Kula, T., Vaimounga, R., 2022. Post-caldera volcanism reveals shallow priming of an intra-ocean arc andesitic caldera: Hunga volcano, Tonga, SW Pacific. Lithos 412-413 (106614). doi: 10.1016/j.lithos.2022.106614
3. Tani, K., Kawabata, H., Chang, Q., Sato, K. & Tatsumi, Y., 2003. Quantitative analyses of silicate rock major and trace elements by X-ray fluorescence spectrometer: Evaluation of analytical precision and sample preparation. *Frontier Research on Earth Evolution* 2, Article 2-16, 8 pp.
