# Supplementary material for: Volcaniclastic density currents explain widespread and diverse seafloor impacts of the 2022 Hunga Volcano eruption
Source: Nat Commun. 2023 Nov 30;14:7881. doi: 10.1038/s41467-023-43607-2 (PMC10689732; doi:10.1038/s41467-023-43607-2)
Supplement: Supplementary file 3 — Description of Additional Supplementary Files [file 41467_2023_43607_MOESM3_ESM.pdf]

Description of Additional Supplementary Files

File Name: Supplementary Data 1
Description: Full sediment, water column, and volcanic ash geochemical data for the plots, interpretations, and figures included within the manuscript and the supplemental information.

File Name: Supplementary Data 2
Description: Raw faunal counts from DTIS video transects of the seafloor that correspond to the data visualizations in Fig 3 and Fig S19.

File Name: Supplementary Movie 1
Description: Video showing the thickness of the volcaniclastic density current over time with black contours indicating bathymetry. (Model 1 from Table S2 with 4 km3 volume of dense fluid). Only part of the domain is shown. The density current can be seen following down the flanks of the Hunga volcano and being steered by the bathymetry. To the south, parts of the flow can be seen overcoming bathymetric barriers and flowing over submarine saddles into the submarine valley where the international cable lay.

File Name: Supplementary Movie 2
Description: Video showing the surface elevation of the overlying water in the two-layer model (Model 1 from Table S2 with 4 km3 volume of dense fluid) i.e., the tsunami waves generated by the movement of the volcaniclastic density current. Only part of the domain is shown. Waves radiate out from the Hunga Volcano and are reflected and refracted by the bathymetry (shown in black contours). White regions show dry land. The resolution of the modelling changes dynamically over the simulation as needed.

File Name: Supplementary Code
Description: Code used for density modelling.
